# ns-μs Time-Resolved Step-Scan FTIR of *ba*_3_ Oxidoreductase from *Thermus thermophilus*: Protonic Connectivity of w941-w946-w927

**DOI:** 10.3390/ijms17101657

**Published:** 2016-09-29

**Authors:** Antonis Nicolaides, Tewfik Soulimane, Constantinos Varotsis

**Affiliations:** 1 Department of Environmental Science and Technology, Cyprus University of Technology, P.O. Box 50329, 3603 Lemesos, Cyprus; ag.nicolaides@edu.cut.ac.cy; 2 Chemical and Environmental Science Department and Materials & Surface Science Institute, University of Limerick, V94 T9PX Limerick, Ireland; tewfik.soulimane@ul.ie

**Keywords:** cytochrome *ba*_3_, ns time-resolved step-scan FTIR, heme-copper oxidoreductases

## Abstract

Time-resolved step-scan FTIR spectroscopy has been employed to probe the dynamics of the *ba*_3_ oxidoreductase from *Thermus thermophilus* in the ns-μs time range and in the pH/pD 6–9 range. The data revealed a pH/pD sensitivity of the D372 residue and of the ring-A propionate of heme *a*_3_. Based on the observed transient changes a model in which the protonic connectivity of w941-w946-927 to the D372 and the ring-A propionate of heme *a*_3_ is described.

## 1. Introduction

The electron and proton transfers in conjunction with the protonic connectivity between the environments sensed by key residues play a vital role in the biological function of proteins [1]. Τhe conformational rigidity of *Thermophilic* enzymes against heat denaturation has attracted the biotechnological research community because of the molecular events associated with enzymatic catalysis. Based on the crystal structure cytochrome *ba*_3_ from *Thermus thermophilus* contains a homodinuclear copper center (Cu_A_), a low-spin heme *b*, and a heme *a*_3_-Cu_B_ center [2,3,4,5,6,7,8,9,10,11,12,13,14,15,16,17,18,19,20,21,22,23,24]. Cytochrome *ba*_3_ catalyzes the reductions of oxygen (O_2_) to water (H_2_O) and of nitric oxide (NO) to nitrous oxide (N_2_O), as well and the oxidation of carbon monoxide (CO) to carbon dioxide (CO_2_) [2,3,4,5,6,7,8,9,10,11,12,13,14,15,16,17,18,19,20,21,22,23,24]. The photolyzed *ba*_3_-CO species is an excellent model for time-resolved spectroscopic studies [7,8,9,13,15,18,24]. In the past, we used time-resolved Raman and step-scan FTIR (TRS^2^-FTIR) spectroscopy to probe the binding of CO to Cu_B_ and the structural changes of the ring-A propionate of heme *a*_3_ and D372 [7,8,9,10]. It was concluded that the *trans*/*cis* isomerization of the ring-D propionate plays a crucial role in controlling the orientations of “docked” CO between the heme rings-A and -D propionates and that the protein environment removes the barrier to the two orientations of CO [10]. The role of the heme *a*_3_-D372-H_2_O site and of ring-A propionate as proton carriers to the H_2_O pool, which is conserved among all structurally-known heme-copper oxidases, were reported [11,16,18,23]. The observation of deprotonated and protonated forms of heme *a*_3_ rings-A and -D propionate and D372 indicated a protonic connectivity between the ring-A propionate, a H_2_O molecule and D372. It was proposed that the environment of the ring-A heme *a*_3_ propionate-D372-H_2_O moiety can contribute to proton motion [18,23].

Time-resolved Raman and step-scan FTIR are powerful structure-sensitive techniques for exploring changes that occur to metal centers and individual amino acids as a result of changes in the ligation state of the metal centers and/or redox and conformational changes induced by the changes in the coordination of the metal centers [24,25,26,27]. The temperature dependency of these changes is expected to give insight into the thermostability of the thermophilic enzymes. Ligand photodissociation can also induce protonation/deprotonation reactions of key residues and the pH dependency of the photodynamic/protonation/deprotonation can contribute towards the elucidation of events not previously reached by other spectroscopic techniques. Furthermore, the detection of protonation/deprotonation of ionizable groups is important towards the elucidation of the proton motions that take place in cytochrome c oxidases. The dynamics of the protein cavities in controlling the motion of O_2_ migration to the binuclear heme Fe-Cu_B_ center is also important towards the elucidation of ligand binding since the enzyme operates at high temperature/low O_2_ concentration. To address these issues the Time Resolved Step-Scan Fourier Transform Infrared (TRS^2^-FTIR) studies of the fully-reduced CO complex in the pH/pD 6–9 range were examined and compared to determine the conformations of the key residue D372 and those of the heme *a*_3_ ring-A propionate. The main goal was to compare the pH/pD results in a time-resolved approach for the protonated and deprotonated forms of *ba*_3_. The effect of H/D exchange and the dynamic behavior of the 1749/1743 and 1723 cm^−1^ modes which have been assigned to ν(COO(H)) of two conformations of the protonated forms of D372, as well as the coupling of the protonic connectivity of w941-w946-w927 to the ring-A propionate of heme *a*_3_ and D372 are discussed.

## 2. Results

**Figure 1:**
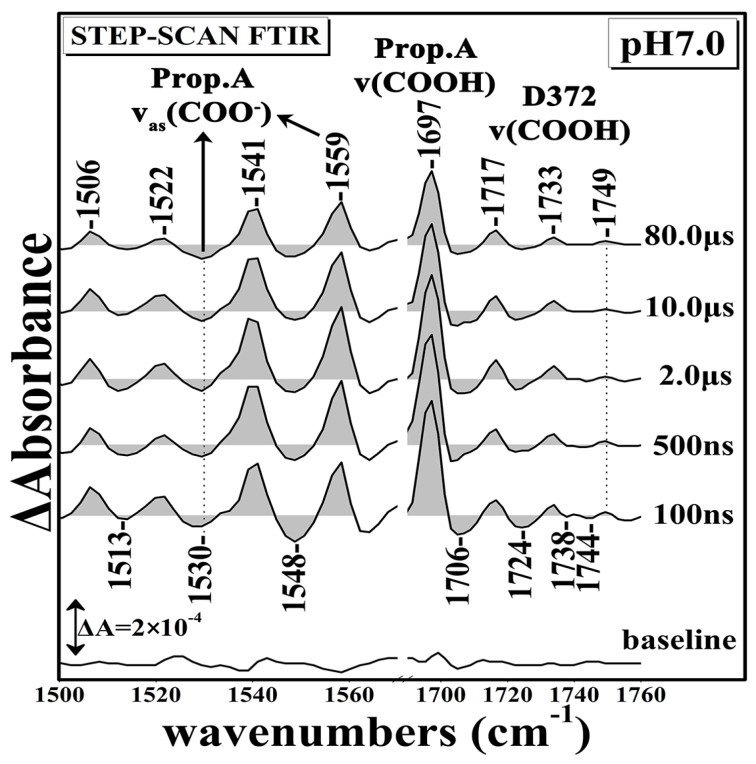
Time Resolved Step-Scan Fourier Transform Infrared (TRS^2^-FTIR) difference spectra of 1500–1760 cm^−1^ region (*t*_d_ = 100–80,000 ns, 4 cm^−1^ spectral resolution) of fully-reduced *ba*_3_-CO subsequent to CO photolysis by a 7 ns 532 nm laser pulse at pH 7.0.

Figure 1 shows the Time Resolved Step-Scan Fourier Transform Infrared (TRS^2^-FTIR) difference spectra (*t*_d_ = 100–80,000 ns, 4 cm^−1^ spectral resolution) of fully-reduced *ba*_3_-CO at pH 7.0 subsequent to CO photolysis by a 7 ns 532 nm laser pulse. At *t*_d_ = 100 ns, the spectra show a peak at 1697 cm^−1^, “W” shape troughs at 1706 and 1724 cm^−1^, and also features at 1717(+), 1733(+), 1738 (−), 1744(−), and 1749(+) cm^−1^. The peak/trough at 1697/1706 cm^−1^ is characteristic of the perturbation of the C=O stretching band exhibiting stronger H-bonding interaction to surrounding groups in the transient spectra that we have assigned to the ring-A propionate of heme *a*_3_. [7]. At *t*_d_ = 500–80,000 ns the 1706 cm^−1^ mode appears as a doublet with intensity and frequency changes. The 1717 and 1733 cm^−1^ modes, which are not exhibiting frequency shifts or intensity changes in the *t*_d_ = 100–80,000 ns range, can be attributed to the C=O mode of either protonated aspartic or glutamic residues which are affected by the induced perturbation of CO photodissociation. The 1749/1738, 1744 cm^−1^ modes have been tentatively assigned to the ν(COO(H)) of two conformations of protonated D372 [7,18]. A broad negative mode at 1548 cm^−1^ is also shown and is tentatively assigned, in agreement with previous work, to originate from the coupled His-Tyr ring mode with large contributions from the C–N of the covalent bond between both ring systems [28]. The 1541 cm^−1^ positive mode appeared as a broad peak and it was attributed to amide II vibrations and remained unchanged in the *t*_d_ = 100–80,000 ns range [29]. Features consisting of a negative peak at 1530 and positive peak at 1559 cm^−1^ are present at *t*_d_ = 100 ns, and were previously assigned to the ν_as_(COO^−^) of the deprotonated form of the ring-A propionate of heme *a*_3_ [11,18,23]. This is evidence that there is equilibrium between the protonated and deprotonated forms of the ring-A propionate of heme *a*_3_. It should be noted that the transient binding of CO to Cu_B_ in *aa*_3_ oxidase is dynamically linked to structural changes around a protonated carboxyl group [30]. Finally, a peak/trough at 1506/1513 cm^−1^ is present and exhibits small intensity changes, but the ratio of the 1506/1513 cm^−1^ modes remained unchanged. This derivative form feature has been attributed to tyrosinate/tyrosine vibrations [31].

**Figure 2:**
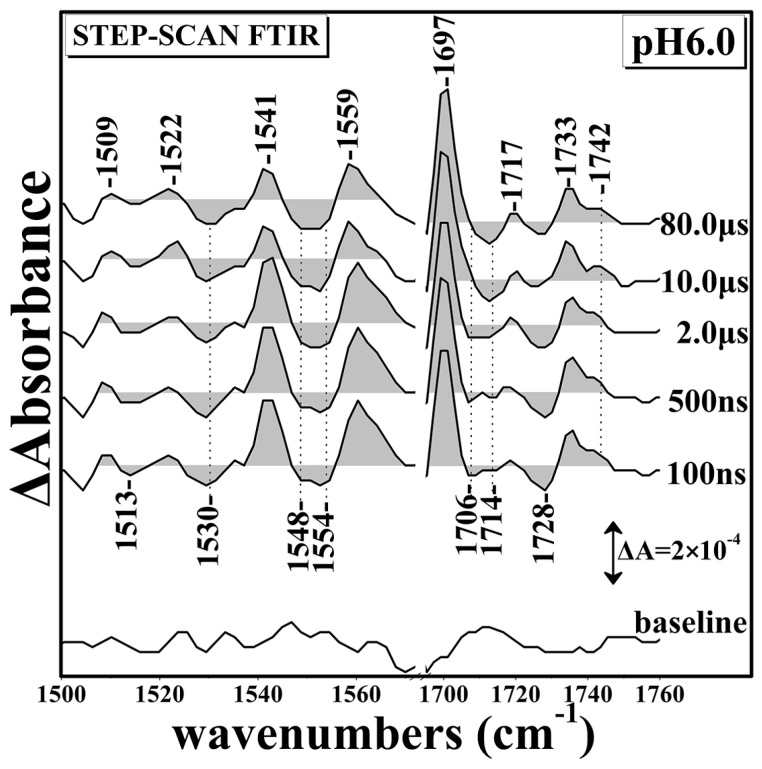
Time Resolved Step-Scan Fourier Transform Infrared (TRS^2^-FTIR) difference spectra of 1500–1760 cm^−1^ region (*t*_d_ = 100–80,000 ns, 4 cm^−1^ spectral resolution) of fully-reduced *ba*_3_-CO subsequent to CO photolysis by a 7 ns 532 nm laser pulse at pH 6.0.

Figure 2 shows the Time Resolved Step-Scan Fourier Transform Infrared (TRS^2^-FTIR) difference spectra of 1500–1760 cm^−1^ region (*t*_d_ = 100–80,000 ns, 4 cm^−1^ spectral resolution) of fully-reduced *ba*_3_-CO at pH 6.0 subsequent to CO photolysis by a 7 ns 532 nm laser pulse. The Time Resolved Step-Scan (TRS^2^) FTIR difference spectra in the 1690–1760 cm^−1^ region show the following changes when compared with those obtained at pH 7. At *t*_d_ = 100 ns, the protonated form of D372 is observed at 1742 cm^−1^, showing a 7 cm^−1^ downshift, which is representative of a weaker C=O bond exhibiting stronger H-bonding interaction to surrounding groups. The 1733 cm^−1^ mode has gained intensity, whereas that of the 1717 cm^−1^ remained the same. The 1697 cm^−1^ mode is not altered in intensity and/or frequency shifts; however, there are two weak negative peaks located at 1706 and 1714 cm^−1^, which at *t*_d_ = 80,000 ns have gained intensity and appeared as a single mode at 1714 cm^−1^. Compared to pH 7, we conclude that there is a pH sensitivity of the protonated forms of D372 and the ring-A propionate of heme *a*_3_. The observed 1728(−), 1733(+), and 1742(+) cm^−1^ modes do not present any intensity changes or frequency shifts in the *t*_d_ = 100–80,000 ns range. Compared to the pH 7 spectra, there is also a frequency shift of the 1723 cm^−1^ mode, which has been attributed to one of the two conformations of D372, to 1728 cm^−1^. This indicates sensitivity upon protonation of the second conformer of D372. The 1559 cm^−1^ mode is broader and the 1541 cm^−1^ mode at pH 6 is similar to that at pH 7. The negative peak at 1548 cm^−1^ observed at pH 7, becomes a doublet with the appearance of a new negative peak at 1554 cm^−1^. The trough at 1530 cm^−1^, which was previously assigned to the deprotonated form of ν_as_(COO^−^) of the ring-A propionate of heme *a*_3_, is present at pH 6 without presenting any changes regarded to the pH alteration.

**Figure 3:**
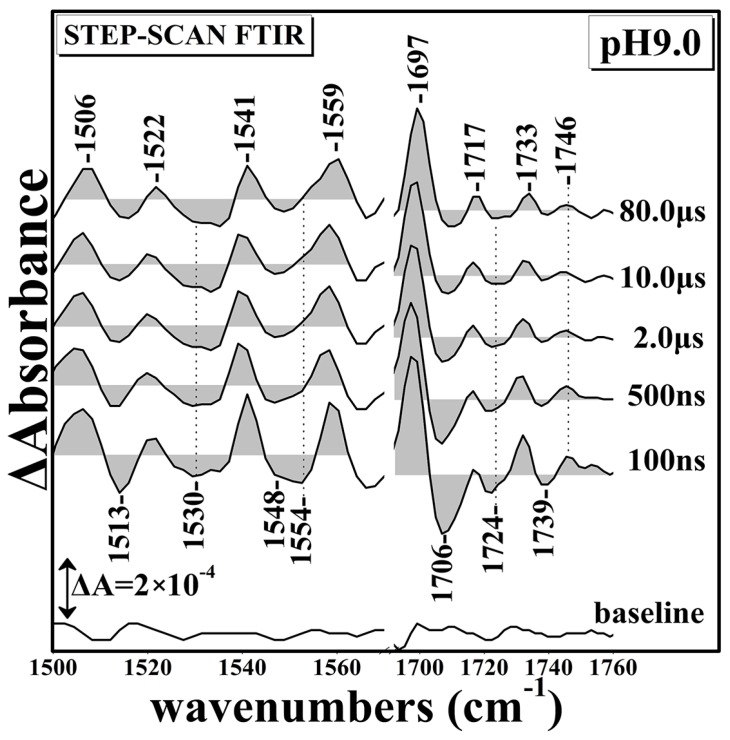
Time Resolved Step-Scan Fourier Transform Infrared (TRS^2^-FTIR) difference spectra of 1500–1760 cm^−1^ region (*t*_d_ = 100–80,000 ns, 4 cm^−1^ spectral resolution) of fully-reduced *ba*_3_-CO subsequent to CO photolysis by a 7 ns 532 nm laser pulse at pH 9.0.

Figure 3 shows the Time Resolved Step-Scan Fourier Transform Infrared (TRS^2^-FTIR) difference spectra of 1500–1760 cm^−1^ region (*t*_d_ = 100–80,000 ns, 4 cm^−1^ spectral resolution) of fully-reduced *ba*_3_-CO at pH 9.0 subsequent to CO photolysis by a 7 ns 532 nm laser pulse. At *t*_d_ = 100 ns, the observed peak/trough feature of 1697/1706 cm^−1^ is similar to that observed at pH 7. This is in contrast to the pH 6 data where two negative peaks at 1706 and 1714 cm^−1^ were observed indicating the pH sensitivity of the ring-A propionate of heme *a*_3_. The protonated form of D372 observed at 1749 cm^−1^ exhibits a 7 cm^−1^ upshift when compared with that observed at pH 6, and a 3 cm^−1^ downshift when compared with that observed at pH 7, confirming the pH sensitivity of the protonated D372. In addition, the negative peak at 1739 cm^−1^, which has been assigned to D372, has gained intensity at *t*_d_ = 100 ns when compared to that at pH 9, but at *t*_d_ = 80 μs has lost almost all of its intensity. This observation indicates that the dynamics of the D372 are linked to the dynamics of the photodissociated CO [7,23]. The deprotonated forms of the ring-A propionate exhibit significant changes as the 1530 cm^−1^ mode appears as a doublet. In addition, at *t*_d_ = 100 ns, there are two trough at 1548 and 1554 cm^−1^. The latter trough loses intensity at times longer than 100 ns and disappears at *t*_d_ = 80 μs, indicating that its behavior is coupled to that of the 1739 cm^−1^ trough.

**Figure 4:**
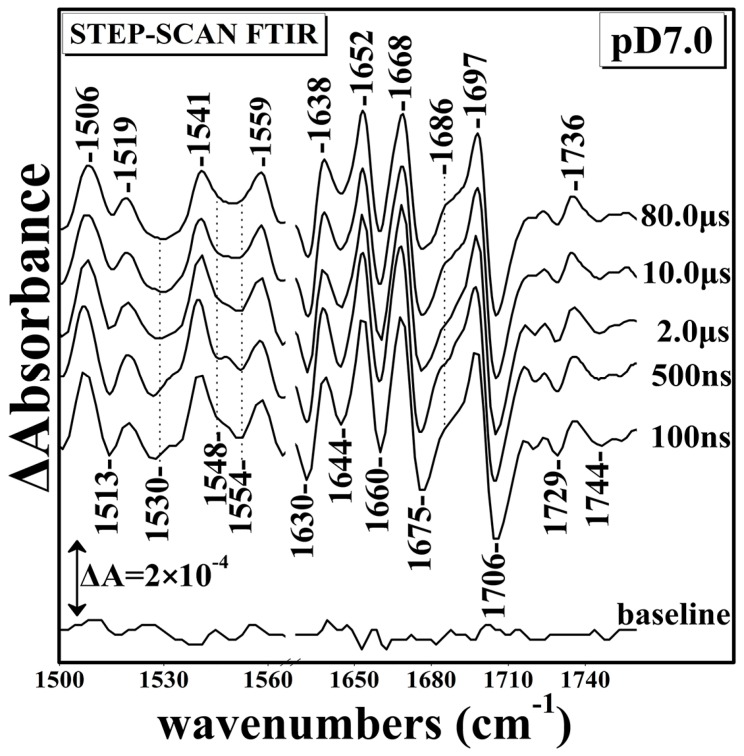
Time Resolved Step-Scan Fourier Transform Infrared (TRS^2^-FTIR) difference spectra of 1500–1760 cm^−1^ region (*t*_d_ = 100–80,000 ns, 4 cm^−1^ spectral resolution) of fully-reduced *ba*_3_-CO subsequent to CO photolysis by a 7 ns 532 nm laser pulse at pD 7.0.

Figure 4 presents the Time Resolved Step-Scan Fourier Transform Infrared (TRS^2^-FTIR) difference spectra of the fully-reduced *ba*_3_-CO complex in D_2_O. The experiments were performed in D_2_O in order to study the behavior of the protein upon H/D exchange. The amide I band arises 80% from the C=O stretching mode of the amide functional group and 20% from C–N stretching [8,9,10,11,12,13]. The protein secondary structure consists of a-helix (1648–1660 cm^−1^), β-sheet (1625–1640 and 1672–1694 cm^−1^), turns (1660–1685 cm^−1^), and unordered structures (1640–1650 cm^−1^) [32,33].

**Figure 5:**
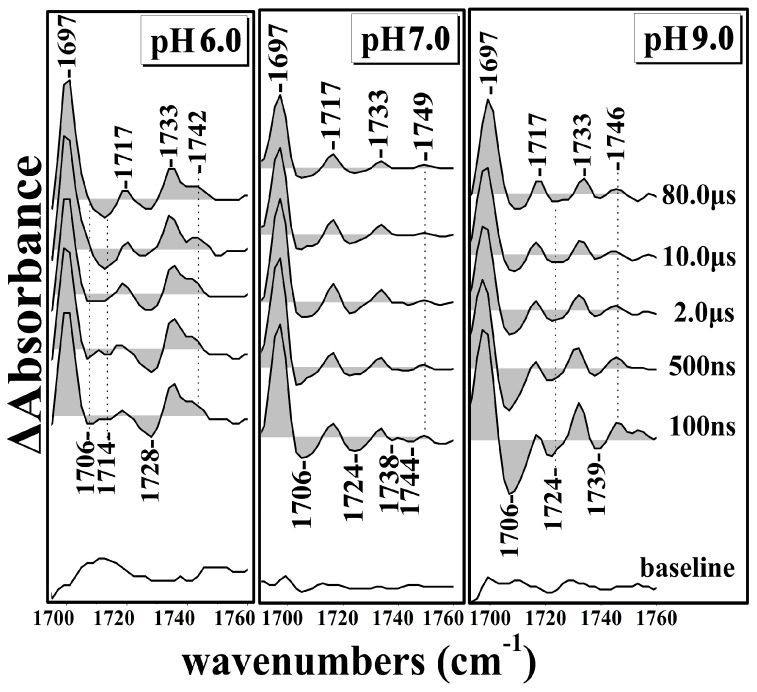
Time Resolved Step-Scan Fourier Transform Infrared (TRS^2^-FTIR) difference spectra of 1690–1760 cm^−1^ region (*t*_d_ = 100–80,000 ns, 4 cm^−1^ spectral resolution) of fully-reduced *ba*_3_-CO subsequent to CO photolysis by a 7 ns 532 nm laser pulse at pH 6.0, 7.0, and 9.0.

Figure 5 presents the pH sensitivity of Propionate A and aspartic acid residue D372. Features at 1736(+)/1744(−), 1729(−), 1697(+)/1706(−), 1686(+), 1668(+)/1675(−), 1652(+)/1660(−), 1638(+)/1644(−), 1630(−), 1559(+), 1541(+)/1548(−) 1519(−)/1527(−), 1535(−), and 1506(+)/1513(−) at 100 ns, subsequent to CO photolysis, remained unchanged in the *t*_d_ = 100–8000 ns range. The 1736(+)/1744(−) and 1729(−) features are slightly pH/pD-dependent since they show small frequency shifts, but the absence of the 1750 cm^−1^ in the pD spectra demonstrates the sensitivity of the protonated form of D372 to pH/pD exchanges. The 1697(+)/1706(−) feature, which has been attributed to the protonated form of the ring-A propionate, is insensitive to pD exchanges. A group of vibrations at 1668(+)/1675(−) are tentatively assigned to protein turns, those at 1652(+)/1660(−) to α-helical group of vibrations and, finally, those at 1638(+)/1644(−), 1630(−) to β-sheet [29,32]. All of the abovementioned vibrations remained unchanged in the *t*_d_ = 100–80,000 ns range. The behavior of all of the vibrational features observed at pD 7 are similar at at pD 6 and pD 9 (Figures S1 and S2).

## 3. Discussion

**Figure 6:**
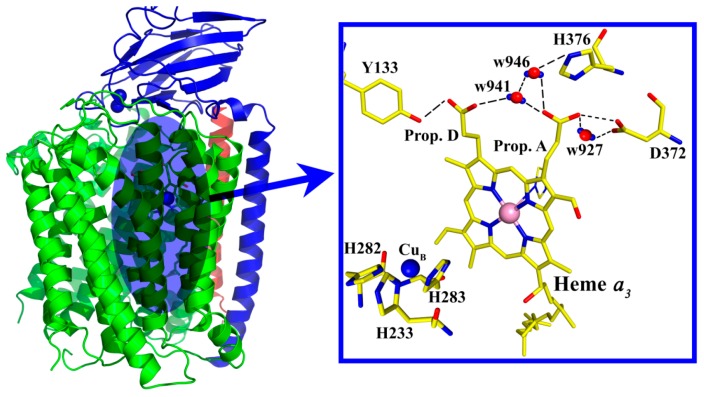
The binuclear heme *a*_3_-Cu_B_ center and region of the heme *a*_3_ propionates of *ba*_3_ oxidoreductase from *Thermus thermophilus* illustrating the residues of interest [6]. Red, yellow and blue colors represent the oxygen, carbon and nitrogen atoms, respectively. The blue sphere represents the Cu_B_ atom. In w941, w946 and w927 the red and blue colors represent the oxygen and hydrogen atoms, respectively. The highlighted water molecules are conserved in heme-copper oxidases [18].

**Figure 7:**
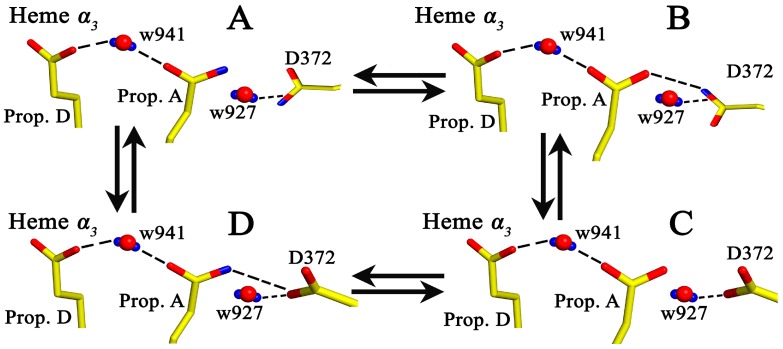
Protonic connectivity between the ring-A propionate of heme *a*_3_, the D372, and the water molecule w927. Blue, red and yellow colors represent protons, oxygen and carbon atoms, respectively. In states **B** and **D**, a single proton is shared between ring-A propionate of heme *a*_3_ and D372, while in state **A**, ring-A propionate of heme *a*_3_ and D372 are protonated and in state **C**, ring-A propionate of heme *a*_3_ and D372 are deprotonated.

The Time Resolved Step-Scan FTIR data have already proven to be a very powerful for understanding the transient changes during protein action. The intensity/frequency changes observed in the TRS^2^-FTIR difference spectra is the result of the perturbation induced by the photodissociation of CO from heme a_3_ and its subsequent binding to Cu_B_ and to the docking site, which consists of the ring-A propionate heme *a*_3_-D372-H_2_O moiety. The presence of protonated/deprotonated forms of D372 and of the ring-A propionate, in association with the dependence of their deprotonated forms on the environment, indicates a protonic connectivity between the D372, the ring-A propionate of heme *a*_3_, and the pair of water molecules w941 and w927. To account for the presence of the observed pH/pD changes and the presence of protonated and deprotonated forms, we present, in Figure 6 and Figure 7, a scheme that includes the ring-A propionate/D372 pair and w927/941. In the oxidative or reductive phase, a proton can be accepted by the ring-A propionate/D372 pair, which influences the release of a proton to the H_2_O pool [34,35,36]. The w941 is not exchangeable; however, it contributes to the dynamics of the ring A-D372-w927. In the scheme, states B and D, in which a single proton is shared between the D372 and the heme *a*_3_ ring A-propionate, can accept a single proton. We propose that this is not operative in the protonated (A) or deprotonated (C) states. We postulate that the observed pH/pD changes in the TRS^2^-FTIR data are due to the exchangeable w927 that provides the H-bonded connection in the local moieties of the D372 and ring-A heme *a*_3_ propionate, and has activation energy for proton motion connecting the ligand docking site with the water pool. Consequently, during the formation of the chemical and pumped H^+^, the H_2_O pool may serve as a primary acceptor for the water molecules. The data reported here indicate that labile protons and w927 are the source of the observed changes to D372, whereas w946-w941-w942, with prop-A-D372 and His-376, form the proton loading site. The observation of H_2_^17^O as a product in the reduction of the O_2_ reaction near H376, which is located in a complex with several crystallographically-detected H_2_O molecules, implies a unique H_2_O exit pathway [34]. At this point it should be noted that the mobility of H_2_O molecules in hydrophobic cavities makes them undetectable by X-ray crystallography. The ability of D_2_O to access the propionate-A-D372 moiety in the pD has been demonstrated by the observed changes to the frequencies of the protonated forms of propionate-A and D372 in the pD 6–9 range. It is suggested that w941/w946 in conjunction with Prop-A-H^+^ acts as the Zundel cation that forms the loading proton site (Figure 6 and Figure 7) [18]. In the absence of water molecules in the binuclear center we conclude that the proton loading site is located in the heme *a*_3_ Prop-A-w946-w941w927-D372 moiety [37].

## 4. Materials and Methods

### 4.1. Sample Preparation

Cytochrome *ba*_3_ was isolated from *Thermus thermophilus* HB8 cells according to previously published procedures. The *ba*_3_ samples were placed in a desired 0.1 M buffer, pH/pD 7.0, HEPES (4-(2-hydroxyethyl) piperazine-1-ethanesulfonic acid), pH/pD 6.0, MES hydrate (2-(*N*-morpholino) ethanesulfonic acid hydrate, 4-morpholineethanesulfonic acid) and pH/pD 9.0, CHES (2-(cyclohexylamino)ethanesulfonic acid). The buffers prepared for the D_2_O experiments were measured assuming pD = pH(observed) + 0.4. The concentration of the samples was determined by UV-VIS measurements performed on a Lambda 25 UV-VIS spectrometer (Perkin Elmer, Italy), using ε_416,ox_ = 152 mM^−1^·cm^−1^, and was ~700 μM. The fully-reduced CO bound form of the enzyme (*ba*_3_-CO) was prepared by using sodium dithionite as a reducing agent and subsequently exposed to 1 atm of CO under anaerobic conditions. The final samples were transferred to an air-tight, sealed FTIR cell, composed by two CaF_2_ windows. The path length was 6 μm for the samples in H_2_^16^O and 15 μm for the samples in D_2_O. The total enzyme volume used for the experiments was ~1.5 mL. The ^12^CO gas was obtained from Messer (Germany) and D_2_O was purchased from Sigma-Aldrich (Taufkirchen, Germany).

### 4.2. ns-μs Time-Resolved Step-Scan FTIR Spectroscopy

**Figure 8:**
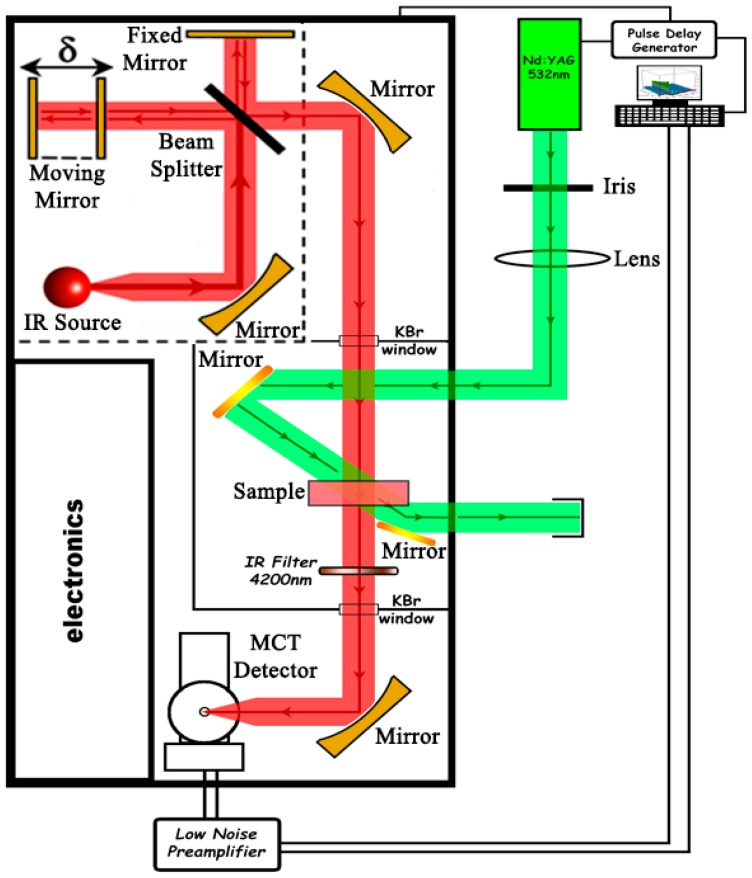
Experimental setup for the ns Time-Resolved Step-Scan Fourier Transform Infrared (ns TRS^2−^FTIR). The red and green arrows represent the infrared beam and the 532 nm photolysis beam, respectively.

The ns-μs Time Resolved Step-Scan Fourier Transform Infrared measurements (TRS^2^-FTIR) were performed on a Vertex 70 v FTIR spectrometer (Bruker, Karlsruhe, Germany) fitted with a liquid nitrogen-cooled fast Mercury-Cadmium-Telluride (MCT) detector (Figure 8). The optical bench was kept under vacuum conditions and the sample compartment was purged with N_2_. The spectral resolution was 4 cm^−1^ and the time resolution was 100 ns. The covered spectral range was 1200–2400 cm^−1^ and an Infrared filter 4200 nm (Spectrogon US INC., Mountain Lakes, NJ, USA) was used. The total number of time slices was 800; 50 of them were taken before the laser triggering and were used as a background reference for the data analysis, and 750 time slices were taken after laser triggering. A 532 nm laser pulse (second harmonic) from a Continuum Minilite Nd-YAG laser (Continuum, San Jose, CA, USA) (7 ns width, 5–8 mJ/pulse, 8 Hz) was used to photolyze the heme *a*_3_-CO complex. Two mirrors were used to direct the 532 nm laser beam inside the spectrometer and through the sample. A Quantum Composers Plus pulse delay generator, Model 9514 (Quantum Composers Inc., Bozeman Montana, MT, USA) was used to synchronize the spectrometer with the laser. A total of 10 coadditions per retardation data point were collected and 35 measurements of single-sided interferograms were collected and averaged in order to improve the S/N ratio. The AC and DC measurements were taken separately using the same sample. The AC signal was amplified by a factor of two using a Model SR560 Low-Noise preamplifier (Stanford research systems, Sunnyvale, CA, USA). The phase from DC measurements was used for phase correction of the AC measurements. The Blackman–Harris three-term apodization function with 32-cm^−1^ phase resolution and the Mertz/No Peak search phase correction algorithm were used. Difference spectra were calculated using ΔA = −log (I_S_/I_R_).

## Supplementary Materials

Supplementary materials can be found at www.mdpi.com/1422-0067/17/10/1657/s1.
