# Supplementary material for: ns-μs Time-Resolved Step-Scan FTIR of ba3 Oxidoreductase from Thermus thermophilus: Protonic Connectivity of w941-w946-w927
Source: Int J Mol Sci. 2016 Sep 29;17(10):1657. doi: 10.3390/ijms17101657 (PMC5085690; doi:10.3390/ijms17101657)
Supplement: Supplementary file 1 [file ijms-17-01657-s001.pdf]

Supplementary Materials: ns-μs Time-Resolved Step-Scan FTIR of *ba*3 Oxidoreductase from *Thermus Thermophilus*: Protonic Connectivity of w941-w946-w927

Antonis Nicolaides, Tewfik Soulimane and Constantinos Varotsis

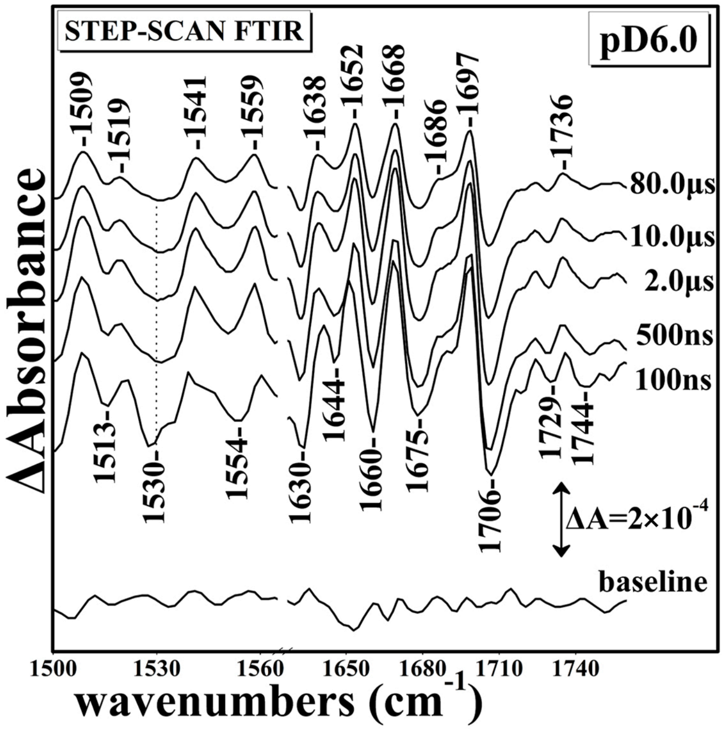

Figure S1. Time Resolved Step-Scan Fourier Transform Infrared (TRS^2-FTIR) difference spectra of 1500–1760 cm^{-1} area (t_d = 100–80,000 ns, 4 cm^{-1} spectral resolution) of fully-reduced *ba*3-CO subsequent to CO photolysis by a 7 ns 532 nm laser pulse, at pD 6.0.

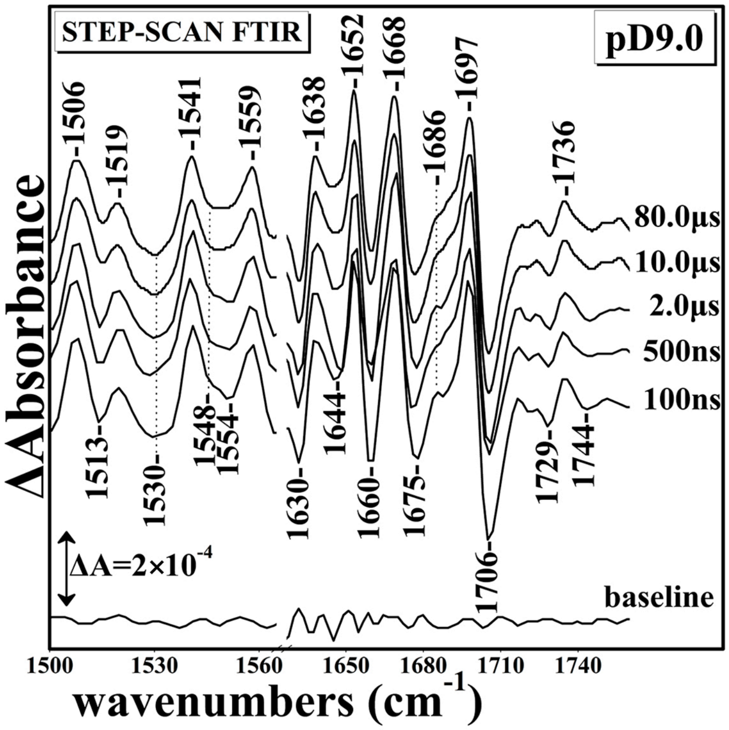

Figure S2. Time Resolved Step-Scan Fourier Transform Infrared (TRS^2-FTIR) difference spectra of 1500–1760 cm^{-1} area (t_d = 100–80,000 ns, 4 cm^{-1} spectral resolution) of fully-reduced *ba*$_3$-CO subsequent to CO photolysis by a 7 ns 532 nm laser pulse at pD 9.0.
